# Assessment of functional decline in stroke patients using 3D deep learning and dynamic functional connectivity based on resting-state fMRI

**DOI:** 10.3389/fneur.2025.1666991

**Published:** 2025-11-13

**Authors:** Yingying Gao, Guojun Xu, Jie Peng, Chengbin Han, Shifei Wu, Minmin Wang, Hewei Wang, Zhiyong Zhao

**Affiliations:** 1 The First Hospital of Xinjiang Production and Construction Group, Aksu, Xinjiang, China; 2 Department of Biomedical Engineering, Children's Hospital, Zhejiang University School of Medicine, National Clinical Research Center for Child Health, Zhejiang University, Hangzhou, China; 3 Department of Rehabilitation, Huashan Hospital, Fudan University, Shanghai, China

**Keywords:** stroke, resting-state functional magnetic resonance imaging, dynamic functional connectivity, ipsilesional primary motor cortex, three-dimensional convolutional neural network

## Abstract

**Introduction:**

This study aimed to develop an automated approach for assessing upper limb (UL) motor impairment severity in stroke patients using a deep learning framework applied to resting-state functional magnetic resonance imaging (rs-fMRI).

**Methods:**

Dynamic functional connectivity (dFC) was computed with the ipsilesional primary motor cortex (M1) as a seed and extracted from rs-fMRI data of 69 stroke patients. These dFC features were used to train a three-dimensional convolutional neural network (3D-CNN) for automatic classification of UL motor impairment severity. Patients were divided into two groups according to UL Fugl-Meyer Assessment (UL-FMA) scores: mild-to-moderate impairment (UL-FMA > 20; *n* = 29, maximum = 66) and severe impairment (0 ≤ UL-FMA ≤ 20; *n* = 40). UL-FMA scores served as labels for supervised learning.

**Results:**

The model achieved a balanced accuracy of 99.8% ± 0.2%, with a specificity of 99.9% ± 0.2% and a sensitivity of 99.7% ± 0.3%. Several brain regions—including the angular gyrus, medial orbitofrontal cortex, dorsolateral superior frontal gyrus, superior parietal lobule, supplementary motor area, thalamus, cerebellum, and middle temporal gyrus—were linked to UL motor impairment severity.

**Discussion:**

These findings demonstrate that a 3D deep learning framework based on dFC features from rs-fMRI enables highly accurate and objective classification of UL motor impairment in stroke patients. This approach may provide a valuable alternative to manual UL-FMA scoring, particularly in clinical settings with limited access to experienced evaluators.

## 1 Introduction

Stroke is a leading cause of long-term disability, with upper limb (UL) motor dysfunction among the most prevalent and debilitating outcomes (1). Such impairments substantially reduce quality of life (2). Although the mechanisms linking stroke lesions to persistent motor deficits remain incompletely understood, neuroimaging studies consistently implicate both structural and functional brain alterations (3–5). Given the heterogeneity of stroke, treatment strategies vary widely and depend critically on the severity of impairment. Accurate assessment of UL motor dysfunction is therefore essential for guiding clinical decision-making, tailoring rehabilitation, and setting realistic recovery expectations for patients and caregivers (6, 7).

The Fugl-Meyer Assessment (FMA) is widely regarded as a reliable and validated tool for evaluating post-stroke motor function (8). It evaluates five domains—motor function, sensation, balance, joint range of motion, and joint pain—with the motor domain most commonly applied to quantify impairment and recovery (9). The UL-FMA specifically examines motor function in the shoulder-arm, wrist, hand, and coordination/speed subsections, progressing from proximal to distal and from synergistic to isolated voluntary movements (9). Each of the 33 items is scored from 0 (absent) to 2 (normal), yielding a maximum of 66 points that reflects full functional capacity (10). Despite its clinical value, the UL-FMA has several limitations: it requires trained therapists, depends on patient cooperation, and is vulnerable to inter-rater variability (9–11). It may also exhibit ceiling effects in higher-functioning patients and is challenging to administer in resource-limited settings or with uncooperative individuals (12, 13). These limitations highlight the need for an objective, automated method of assessing UL motor impairment severity.

Resting-state functional magnetic resonance imaging (rs-fMRI) offers a complementary neuroimaging approach to evaluate post-stroke motor dysfunction, particularly through analyses of functional connectivity (FC) within motor-related regions. Prior work has demonstrated post-stroke FC reorganization involving the ipsilesional primary M1 and regions such as the thalamus, SMA, middle frontal gyrus, and cerebellum (3, 14–17). Interhemispheric M1 connectivity has also been positively correlated with motor performance during the subacute phase of stroke (12, 18), suggesting that ipsilesional M1 FC may represent a biomarker of motor impairment. Traditional rs-fMRI analyses, however, often assume that FC remains stable during the scanning. Emerging evidence indicates that brain connectivity is inherently dynamic (19–21). Dynamic FC (DFC) captures temporal fluctuations in FC, providing a richer characterization of brain activity than static approaches (21). For example, dFC between the ipsilesional M1 and contralesional precentral gyrus has been negatively correlated with FMA scores in stroke patients (22). Using sliding time-window methods, dFC generates time-resolved connectivity matrices that can be clustered to identify distinct connectivity states and their temporal properties (23).

Deep learning has become a powerful tool in medical image analysis, offering automated feature extraction and classification capabilities that surpass traditional approaches (24, 25). In stroke research, deep learning holds promise for minimizing reliance on expert evaluation in the classification of motor impairment severity (26, 27). Unlike conventional algorithms that require manual feature engineering and operate on one-dimensional data, deep learning models—particularly three-dimensional convolutional neural networks (3D-CNNs)—can directly process volumetric neuroimaging data (28, 29). 3D-CNNs have shown strong performance in various medical imaging tasks, excelling in classification and pattern recognition within supervised frameworks (24, 26, 30). Although their application to brain imaging remains relatively limited due to computational demands, dFC-derived features from the ipsilesional M1 can reduce data dimensionality while retaining spatiotemporal patterns relevant to stroke severity.

In this study, we propose a novel framework for automated dichotomous classification of UL motor impairment (mild/moderate vs. severe) in stroke patients. We hypothesize that dFC features derived from rs-fMRI, particularly those involving the ipsilesional M1, can serve as effective biomarkers of motor impairment severity. By leveraging a 3D-CNN architecture, our goal is to enable accurate, objective classification and provide a viable alternative to conventional clinical assessment tools such as the UL-FMA.

## 2 Methods

### 2.1 Subjects

A total of 69 patients in the chronic phase of subcortical stroke were recruited from Huashan Hospital and underwent neuroimaging at the Shanghai Key Laboratory of Magnetic Resonance. Inclusion criteria were: (1) first-ever subcortical stroke; (2) duration of illness > 3 months; (3) Montreal Cognitive Assessment (MoCA) score > 23, indicating preserved global cognition; and (4) right-handedness. Exclusion criteria were: (1) contraindications to MRI; (2) bilateral stroke lesions; and (3) comorbid neurological or psychiatric disorders unrelated to stroke. Cognitive function was assessed using the MoCA, administered by an experienced therapist at admission. UL motor function was evaluated using the UL-FMA scale. The study was conducted in accordance with the Declaration of Helsinki, approved by the Review Board of Ethics Committee of Huashan Hospital, and registered at the Chinese Clinical Trial Registry (ChiCTR-TRC-08003005). Written informed consent was obtained from all participants.

To investigate resting-state functional connectivity alterations associated with UL motor impairment severity, patients were categorized into two groups based on their UL-FMA scores: those scoring > 20 were classified as having mild-to-moderate impairment (Mild/Moderate Stroke Patients, MSP; *n* = 29), and those with scores ≤ 20 as having severe impairment (Severe Stroke Patients, SSP; *n* = 40) (9, 13, 31). Demographic comparisons between groups were performed using SPSS version 23.0 for Windows.

### 2.2 MRI data acquisition

All imaging was conducted on a 3.0-T Siemens MRI scanner (Erlangen, Germany) at the Shanghai Key Laboratory of Magnetic Resonance. High-resolution T1-weighted structural images were acquired using a magnetization-prepared rapid gradient echo (MPRAGE) sequence with 192 sagittal slices and the following parameters: repetition time (TR) = 1900 ms; echo time (TE) = 3.42 ms; inversion time (TI) = 900 ms; flip angle = 9°; field of view (FOV) = 240 × 240 mm^2^; matrix = 256 × 256; slice thickness/gap = 1/0.5 mm. T2-weighted images for lesion localization were obtained with a turbo spin-echo sequence (30 axial slices; TR = 6,000 ms; TE = 93 ms; FOV = 220 × 220 mm^2^; flip angle = 120°; matrix = 320 × 320; slice thickness/gap = 5/0 mm). Rs-fMRI data were collected using an echo-planar imaging (EPI) sequence (30 axial slices; TR = 2000 ms; TE = 30 ms; flip angle = 90°; FOV = 220 × 220 mm^2^; matrix = 64 × 64; slice thickness/gap = 4/0.8 mm; 240 volumes). The total acquisition time was 8 min 6 s, including a 6-s dummy scan to ensure magnetization equilibrium. During rs-fMRI scanning, participants were instructed to remain still, keep their eyes closed, stay awake, and avoid focused thought.

### 2.3 Lesion analysis

**Figure 1:**
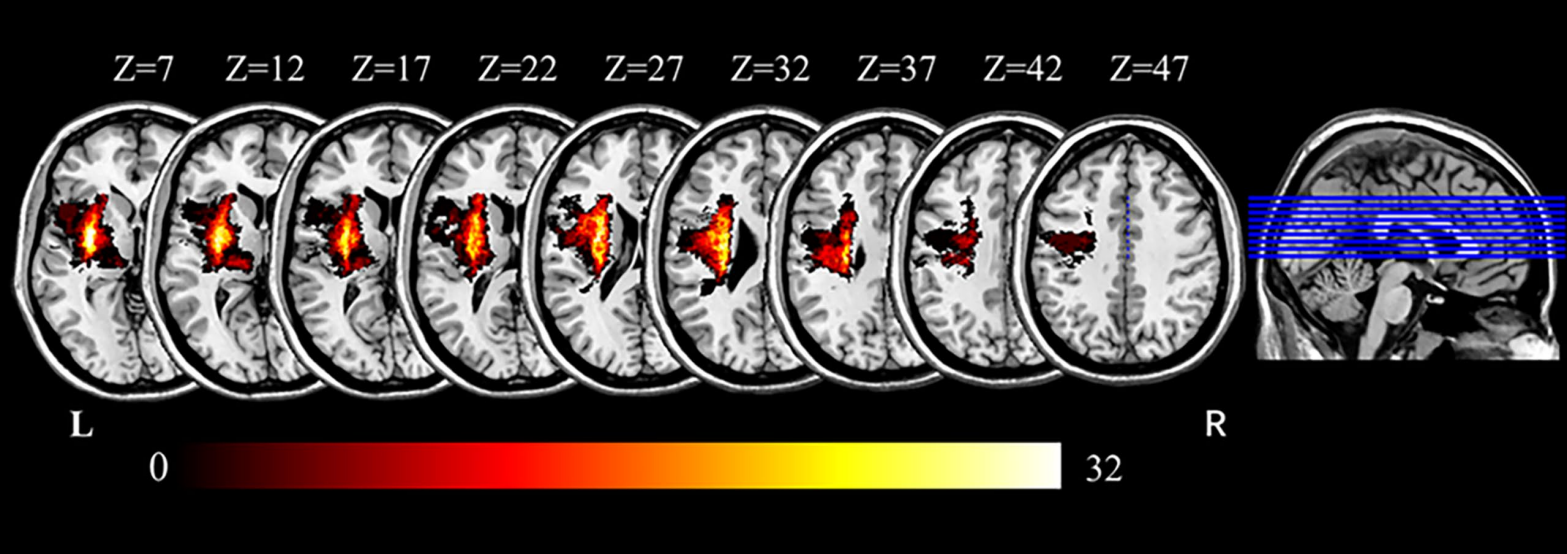
Overlap map of stroke lesions across all participants. Color intensity indicates the number of patients with overlapping lesions at each voxel. Axial slices are displayed along the z-axis from slice 7 to slice 47 in Montreal Neurological Institute (MNI) space (MNI, Montreal Neurological Institute; R, right; L, left).

Lesion volumes were manually delineated on T1- and T2-weighted images by two experienced radiologists using MRIcron software^1^. Lesions were outlined slice by slice for each patient, with T1-weighted images used for primary outlining and T2-weighted images used for validation. To standardize lesion laterality, all images were mirrored so that lesions were represented on the left hemisphere (Figure 1). The resulting lesion masks were used for visualization, lesion volume calculation, and exclusion of intra-lesion voxels during dFC analysis. Notably, lesion volume was not significantly correlated with UL motor function, as measured by UL-FMA scores (Pearson’s *r* = −0.208, *p* = 0.087), suggesting that stroke size alone did not account for impairment severity in this cohort.

### 2.4 fMRI data preprocessing

Image preprocessing and analysis were performed using Statistical Parametric Mapping (SPM12^2^) and the Data Processing Assistant for RS-fMRI (DPABI^3^). For patients with right-hemisphere lesions, images were flipped along the midsagittal plane to standardize orientation, designating the left hemisphere as ipsilesional and the right as contralesional for all participants. Preprocessing steps were as follows: (1) The first 10 volumes of each functional scan were discarded to allow for magnetization equilibrium and participant adaptation. The remaining 230 volumes were corrected for slice timing, and head motion. No subject was excluded, as none exceeded the motion threshold of 2.0 mm translation or 2.0° rotation; (2) Linear trends, mean white matter and cerebrospinal fluid (CSF) signals, and 24 motion parameters (translations, rotations, their temporal derivatives, and quadratic terms) were regressed out of each voxel time series. Global signal regression was not applied to preserve correlation matrix structure, particularly for sliding-window analyses (32, 33); (3) Functional images were spatially normalized to Montreal Neurological Institute (MNI) space using each patient’s lesion mask and the DARTEL (Diffeomorphic Anatomical Registration Through Exponentiated Lie Algebra) algorithm (34). Images were resampled to 3 × 3 × 3 mm^3^ voxels using the DARTEL-derived deformation fields; (4) Time-series data were spatially smoothed with a 6 mm full-width at half-maximum (FWHM) Gaussian kernel, linearly detrended, and band-pass filtered (0.01–0.1 Hz) to reduce physiological and low-frequency noise.

### 2.5 Dynamic functional connectivity

**Figure 2:**
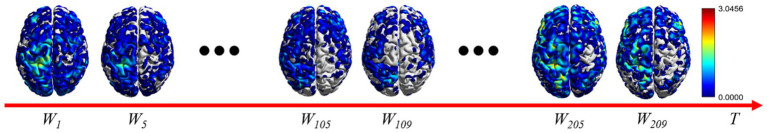
DFC maps across different sliding windows for a representative participant. Each map illustrates the time-varying connectivity between the left primary motor cortex (M1) and other brain regions. The color bar denotes Fisher’s Z-transformed correlation coefficients (T, time; W, window).

DFC analysis was conducted using the Temporal Dynamic Analysis (TDA) module within the DPABI toolbox (35). A sliding-window approach was applied to capture temporal fluctuations in connectivity between the left primary M1 and all other brain voxels (36). The left M1 seed was defined at MNI coordinates (−38, −22, 56), consistent with prior studies implicating this region in UL motor function after stroke (3, 12, 16, 37, 38). Each sliding window covered 22 TRs (44 s) and shifted by 1 TR (2 s), enabling fine-grained tracking of temporal dynamics (19). This window length was selected according to the lower limit of the frequency spectrum (<0.5/*f*_lower_ = 50 s; here, *f*_lower_ = 0.01 Hz), ensuring reliable estimation while preserving sensitivity to short-term fluctuations (39). A 22-TR window therefore represents a balance between temporal resolution and estimation stability, consistent with prior literature (16, 19, 39). From the 230 rs-fMRI volumes, each participant contributed 209 overlapping sliding windows. Within each window, Pearson correlation coefficients were calculated between the left M1 time series and all other brain voxels, generating a dynamic series of whole-brain correlation maps (Figure 2). Fisher’s r-to-z transformation was then applied to improve normality of the correlation values (40).

### 2.6 Feature extraction

The 3D volumetric functional connectivity maps were used as inputs to a 3D-CNN to classify participants into either the MSP or SSP group. Consistent with prior neuroimaging studies, no standard data augmentation techniques were applied, as introducing synthetic data could bias the training process. Rs-fMRI signals are particularly sensitive; even a 3 mm spatial shift can substantially alter their interpretation. For this reason, conventional augmentation strategies are generally discouraged in the neuroimaging community (24, 26, 28).

### 2.7 Deep learning and 3D-CNN framework

**Figure 3:**
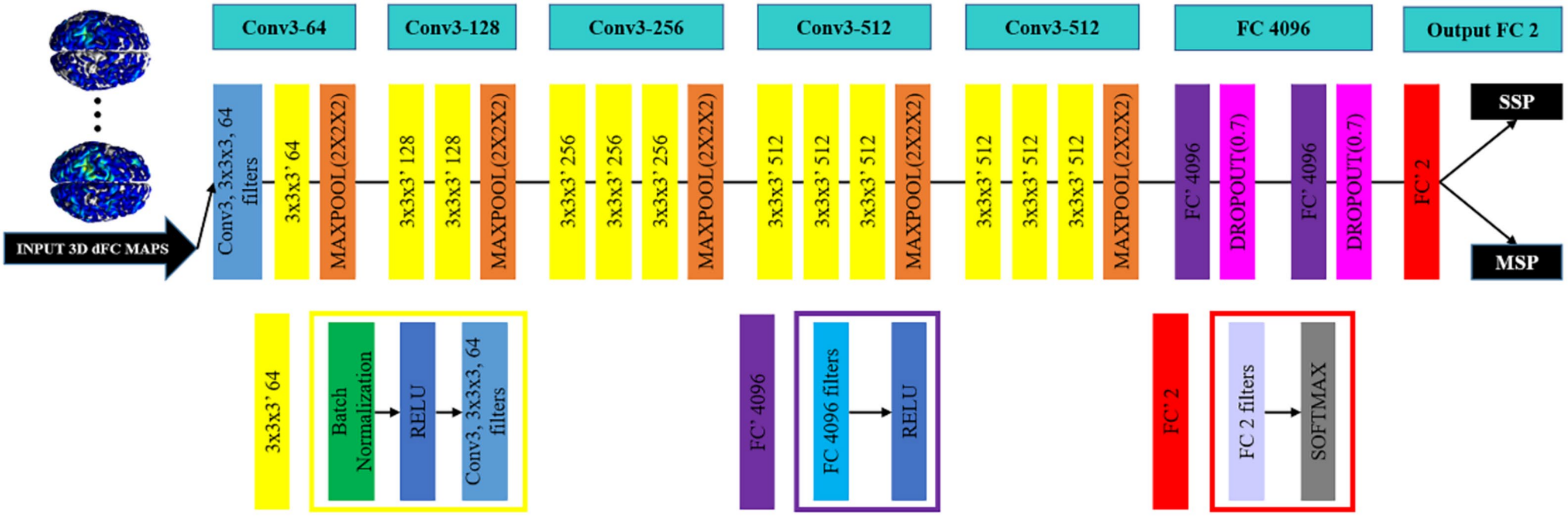
VGG-Net-based 3D-CNN architecture. SSP, severely stroke patients; MSP, mild stroke patients; dFC, dynamic functional connectivity.

A 3D-CNN–based deep learning framework was developed for participant classification. The model was implemented in MATLAB 2019b with GPU acceleration on an NVIDIA Quadro RTX 5000. Training employed the Adam optimizer (41) with an initial learning rate of 0.001, which was reduced by half every 10 epochs. Each epoch consisted of 32-sample mini-batches over 50 epochs in total. An epsilon value of 0.001 was applied, and cross-entropy loss was used as the cost function. To mitigate overfitting, 10-fold cross-validation was performed to estimate mean classification accuracy at the dFC level. For each fold, 10% of the dFC maps were reserved for testing, while the remaining data were split into training (80%) and validation (10%) sets. The network was based on a modified VGG-Net architecture (42), incorporating batch normalization in convolutional layers and Rectified Linear Unit (ReLU) activation functions. A dropout rate of 0.7 was applied to the fully connected layers to enhance generalization. Weight initialization followed the method proposed by He et al. (43), using a zero-mean Gaussian distribution with a standard deviation scaled to network depth. Hyperparameters—including learning rate, epsilon, dropout rate, batch size, and number of epochs—were optimized based on prior work (24, 26). Specifically, epsilon was tuned in the range [0.1:0.05:1], learning rate across a logarithmic scale [1, 0.1, 0.01, 0.001, 0.0001, 0.00001], and dropout rate between [0.1:0.05:1]. Batch size was optimized using the maximum available GPU memory, while the number of epochs was tuned within [10:1:200]. The complete 3D-CNN architecture is shown in Figure 3 and detailed in Table 1.

**Table 1:** Details of the three-dimensional convolutional neural network (3D-CNN) architecture.

Layer	Feature map	Stride	Kernel	Activation structure
3D Input	61 × 73 × 61			
Convolution	64	1x1x1	3x3x3	Conv
Convolution	64	1x1x1	3x3x3	Batchnorm + ReLU + Conv
Maxpool		2x2x2	2x2x2	
Convolution	128	1x1x1	3x3x3	Batchnorm + ReLU + Conv
Convolution	128	1x1x1	3x3x3	Batchnorm + ReLU + Conv
Maxpool		2x2x2	2x2x2	
Convolution	256	1x1x1	3x3x3	Batchnorm + ReLU + Conv
Convolution	256	1x1x1	3x3x3	Batchnorm + ReLU + Conv
Convolution	256	1x1x1	3x3x3	Batchnorm + ReLU + Conv
Maxpool		2x2x2	2x2x2	
Convolution	512	1x1x1	3x3x3	Batchnorm + ReLU + Conv
Convolution	512	1x1x1	3x3x3	Batchnorm + ReLU + Conv
Convolution	512	1x1x1	3x3x3	Batchnorm + ReLU + Conv
Maxpool		2x2x2	2x2x2	
Convolution	512	1x1x1	3x3x3	Batchnorm + ReLU + Conv
Convolution	512	1x1x1	3x3x3	Batchnorm + ReLU + Conv
Convolution	512	1x1x1	3x3x3	Batchnorm + ReLU + Conv
Maxpool		1x1x1	2x2x2	
Fully Connected	4,096	Dropout Rate o.7		ReLU
Fully Connected	4,096	Dropout Rate o.7		ReLU
Output				
Fully Connected	2			ReLU
Softmax				
Classification Layer		Argmax		

### 2.8 Dynamic states and clustering

To examine the contribution of common functional connectivity to deep learning classification and to better understand how deep neural networks iteratively optimize classification weights (24), we analyzed dFC between the ipsilesional M1 and other brain regions across individual sliding windows. Because multiple pairwise comparisons can introduce statistical bias, we applied a k-means clustering algorithm to dFC maps from all participants to identify distinct connectivity states at rest and to assess intra-individual variability. Following previous studies (44), L1 distance was chosen as the similarity metric due to its robustness in high-dimensional data. The clustering procedure consisted of two main steps. First, exemplar datasets were selected based on local maxima in functional connectivity variance across windows, consistent with prior work (19, 23). Second, rather than relying on conventional criteria such as the elbow method or Silhouette index—which may not guarantee that each participant is represented across multiple states—we determined the optimal number of clusters (k) by evaluating the participation rate (PR) of subjects across candidate solutions (19, 23). Cluster solutions were examined for k values ranging from 2 to 8. A three-cluster solution (*k* = 3) was selected because it yielded an adequate distribution of subject participation (state 1: PR = 1/69; state 2: PR = 68/69; state 3: PR = 69/69). States 2 and 3 were retained for further analysis, while state 1 was excluded due to insufficient subject representation, which precluded statistically meaningful comparisons.

### 2.9 Significance testing

To evaluate the statistical significance of our results, we performed a permutation test on the 3D-CNN classification accuracies, followed by a two-sample *t*-test to assess between-group differences. For the classification analysis, test data labels were randomly shuffled 1,000 times within each of the 10 cross-validation folds to estimate the probability of achieving an accuracy greater than that obtained with the true labels. Between-group differences in dFC (MSP vs. SSP) were tested using two-sample *t*-tests, with age, sex, and duration of illness included as covariates. A significance threshold of uncorrected *p* < 0.05 was adopted.

## 3 Results

### 3.1 Demographic characteristics

No significant differences were observed between the MSP and SSP groups in age (MSP: 54.3 ± 11.95 years; SSP: 51.28 ± 10.73 years; *p* = 0.392, two-sample *t*-test), sex distribution (MSP: 32 males; SSP: 24 males; *p* = 0.513, *χ*^2^ test), duration of illness (MSP: 8.01 ± 6.5 months; SSP: 9.40 ± 7.10 months; *p* = 0.410, two-sample *t*-test), or lesion volume (MSP: 6.7 ± 4.73 mL; SSP: 4.97 ± 4.70 mL; *p* = 0.513, two-sample *t*-test). As expected, UL-FMA scores were significantly different, with higher scores in the MSP group compared to the SSP group (31.4 ± 7.84 vs. 8.85 ± 4.49; *p* < 0.0001). Detailed demographic and clinical characteristics for all participants, including healthy controls (HCs), are presented in Table 2.

**Table 2:** Demographic, clinical and structural MRI data of the participants.

	SSP (*n* = 40) Mean ± std	MSP (*n* = 29) Mean ± std	SSP vs. MSP *p*-value
Age (years)^a^	54.03 ± 11.95	51.28 ± 10.73	0.392
Sex (male: female)	8/32	5/24	0.513
Lesion volume (ml)^a^	6.7 ± 4.73	4.97 ± 4.70	0.137
Duration of illness (month)^a^	8.01 ± 6.52	9.40 ± 7.10	0.410
Lesion side (left: right)	22/18	15/14	0.490
UL-FMA^a^	8.85 ± 4.49	31.4 ± 7.84	0.0001

^a^Independent *t*-test. UL-FMA, up limb Fugl-Meyer assessment; SSP, severely stroke patients; MSP, mild/moderate stroke patients.

### 3.2 Classification

The 3D-CNN achieved high classification performance under 10-fold cross-validation. To mitigate potential bias from class imbalance, balanced accuracy was reported alongside other evaluation metrics. The mean performance across folds was as follows: training accuracy = 99.23%, test accuracy = 99.80%, specificity = 99.86%, sensitivity = 99.74%, F-score = 99.78%, and balanced accuracy = 99.80% (Table 3).

**Table 3:** Classification accuracy using 10-fold cross-validation.

Fold	Train ACC (%)	Test ACC (%)	*p*-value	AUC	Spec (%)	Sen (%)	*F*-score (%)	BAC (%)
1	99.72	99.45	<0.001	0.9994	99.4	99.51	99.34	99.45
2	99.96	99.65	<0.001	0.9999	99.88	99.34	99.59	99.61
3	99.72	99.69	<0.001	0.9999	99.76	99.67	99.67	99.71
4	100	99.93	<0.001	1	99.88	100	99.91	99.94
5	100	100	<0.001	1	100	100	100	100
6	99.99	99.86	<0.001	1	99.88	99.84	99.84	99.86
7	99.95	100	<0.001	1	100	100	100	100
8	99.92	99.51	<0.001	0.9999	99.76	99.17	99.42	99.47
9	100	99.93	<0.001	1	100	99.83	99.92	99.92
10	99.97	100	<0.001	1	100	100	100	100
Mean ± SD	99.23 ± 0.10	99.80 ± 0.20		0.9999 ± 0.0002	99.86 ± 0.18	99.74 ± 0.29	99.78 ± 0.24	99.80 ± 0.21

Test ACC, test accuracy; Train ACC, train accuracy; Spec, specificity; Sen, sensitivity; BAC, balanced accuracy.

Statistical significance was confirmed using permutation testing. A threshold of *p* = 0.001 was applied across all folds, and permutation tests consistently yielded *p* < 0.001, indicating that the observed classification performance was highly unlikely to arise by chance. Fold-wise permutation-derived *p*-values are presented in Table 3.

### 3.3 Clinical significance

**Figure 4:**
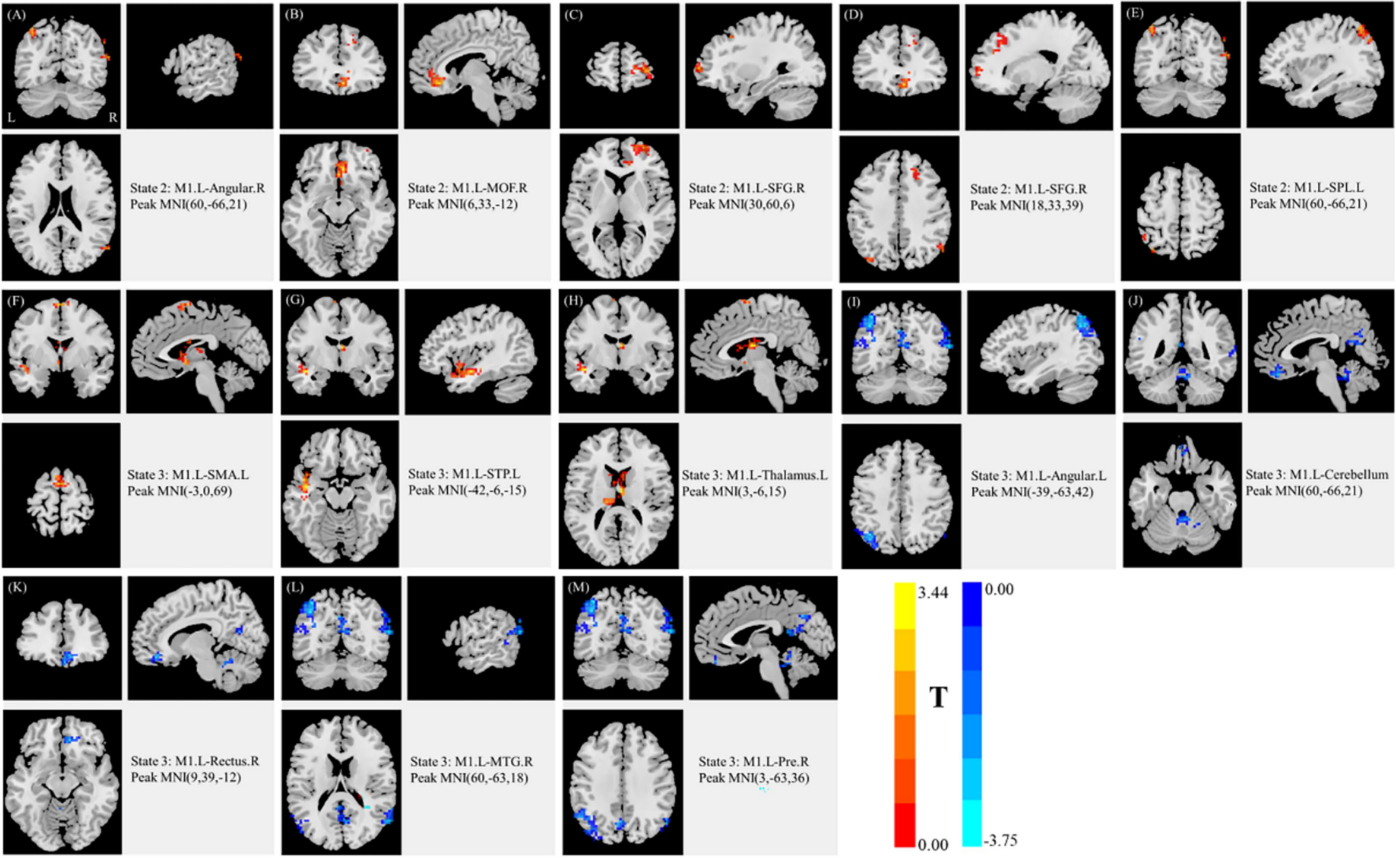
Discriminative features of selected states. **(A–E)** correspond to state 2, and **(F–M)** correspond to state 3, respectively. L, left; R, right; Angular, angular gyrus; MOF, medial orbitofrontal cortex; SFG, dorsolateral superior frontal gyrus; SPL, superior parietal lobule; SMA, supplementary motor area; STP, superior temporal pole; Thalamus, thalamus; cerebellum, cerebellum; Rectus, gyrus rectus; MTG, middle temporal gyrus; Pre, precuneus; MNI, Montreal Neurological Institute. Two-tailed two-sample *t*-test, *p* < 0.05 (|T| > 2.0), cluster size ≥ 50, uncorrected. Red indicates regions where functional connectivity was higher in MSP than in SSP; blue indicates the opposite (MSP, mild stroke patients; SSP, severe stroke patients).

Discriminative brain regions contributing to the deep learning framework were identified using two-sample *t*-tests across dynamic states 2 and 3. Significant regions included the angular gyrus (Angular), MOF, SFG, SPL, SMA, superior temporal pole (STP), thalamus, cerebellum, gyrus rectus, middle temporal gyrus (MTG), and precuneus. Uncorrected *t*-values indicated group differences in functional connectivity: indices A-E corresponded to state 2 (*t* = 2.64–3.37), and indices F-M corresponded to state 3 (*t* = 2.88–3.75) (Table 4 and Figure 4). In Figure 4, red shading denotes regions where functional connectivity was higher in the MSP group compared to the SSP group, while blue shading indicates higher connectivity in SSP relative to MSP.

**Table 4:** Statistical analysis of each state.

Index	State	Group comparisons	Brain regions (AAL)	Peak MNI coordinates			Cluster voxels	Peak T values
				*X*	*Y*	*Z*		
A	2	MSP > SSP	Angular. R	60	−66	21	52	2.9
B	2	MSP > SSP	MOF. R	6	33	−12	161	3.37
C	2	MSP > SSP	SFG. R	30	60	6	86	3.01
D	2	MSP > SSP	SFG. R	18	33	39	58	2.64
E	2	MSP > SSP	SPL. L	−36	−66	57	103	2.92
F	3	MSP > SSP	SMA. L	−3	0	69	50	2.89
G	3	MSP > SSP	STP. L	−42	−6	−15	127	3.136
H	3	MSP > SSP	Thalamus. L	3	−6	15	126	3.15
I	3	MSP < SSP	Angular. L	−39	−63	42	471	3.75
J	3	MSP < SSP	Cerebellum	3	−45	−24	77	2.88
K	3	MSP < SSP	Rectus. R	9	39	−12	77	3.31
L	3	MSP < SSP	MTG. R	60	−63	18	129	3.52
M	3	MSP < SSP	Pre. L	−3	−63	36	102	3.25

L, left; R, right; Angular, angular gyrus; MOF, medial orbitofrontal cortex; SFG, dorsolateral superior frontal gyrus; SPL, superior parietal lobule; SMA, supplementary motor area; STP, superior temporal pole; Thalamus, thalamus; Cerebellum, cerebellum; Rectus, gyrus rectus; MTG, middle temporal gyrus; Pre, precuneus; MNI, Montreal Neurological Institute. Two-tailed two-sample *t*-test, *p* < 0.05 (|T| > 2.0), cluster size ≥ 50, uncorrected.

## 4 Discussion

Using the 3D-CNN framework, we achieved high classification performance, with a mean balanced accuracy of 99.80%, specificity of 99.86%, and sensitivity of 99.74%. Accurate assessment of UL motor impairment in stroke is critical for guiding treatment decisions, highlighting the clinical relevance of automated classification methods. To identify features driving classification, we examined state-specific alterations in resting-state dFC of the ipsilesional primary M1. Comparisons between MSP and SSP groups across two connectivity states revealed several discriminative regions—including the angular gyrus, MOF, SFG, SPL, SMA, STP, thalamus, cerebellum, gyrus rectus, and MTG—which were subsequently integrated into the deep learning framework. These findings support our hypothesis that ipsilesional M1-derived dFC can serve as a functional biomarker for classifying UL motor impairment severity using 3D-CNN.

Previous studies have demonstrated the feasibility of using machine learning with multimodal neuroimaging data, including structural MRI and clinical assessments, for automated classification and outcome prediction in stroke (24, 26, 28, 29, 45–48). Several studies successfully predicted UL motor impairment using these approaches (45–48). While structural MRI can distinguish MSP from SSP, it is often limited by inter-individual variability and may fail to capture subtle changes, particularly when lesion volume, shape, or corticospinal tract damage exceeds certain thresholds (47). In contrast, rs-fMRI provides a dynamic and individualized assessment, with shorter acquisition times that enhance clinical efficiency.

Prior research has shown that interventions such as repetitive transcranial magnetic stimulation can improve FMA scores in patients with severe UL impairment by increasing excitability in the ipsilesional M1 (7). Additionally, dFC between the ipsilesional M1 and contralesional precentral and middle frontal gyri has been negatively correlated with FMA scores (22), supporting the potential of M1-derived dFC as a predictor of motor impairment severity. To our knowledge, this study is the first to use dFC maps in combination with a 3D-CNN framework to classify stroke patients into mild/moderate versus severe UL-FMA groups, representing a novel contribution to automated stroke severity assessment.

In our 10-fold cross-validation, the 3D-CNN achieved a mean test accuracy of 99.80%. Although deep neural networks iteratively optimize classification weights (24–26, 28), ranking the contribution of individual dFC features remains challenging without feature selection. To address this, we analyzed dFC differences between MSP and SSP across states using k-means clustering. Statistical analysis revealed significant connectivity differences between the ipsilesional M1 and 13 other regions (*p* < 0.05, cluster size ≥ 50, uncorrected). Prior work has implicated the contralesional cerebellum (46), SMA (49), and contralesional parietal cortex (50) in motor recovery or stroke severity. Our findings are largely consistent with these reports, while also revealing increased connectivity in the ipsilesional SMA and SPL, which may reflect specific resting-state configurations rather than averaged connectivity patterns (22). Notably, significant connectivity differences were observed in the contralesional angular gyrus, MOF, SFG, gyrus rectus, MTG, as well as the ipsilesional STP, thalamus, angular gyrus, precuneus, and cerebellum, consistent with widespread functional plasticity following stroke (12, 23, 37). These regions likely contributed substantially to classifier performance, although additional regions may also play roles through subtle, weighted patterns learned by the network.

Consistent with previous findings, reduced connectivity between the ipsilesional M1 and contralesional hemisphere is observed after unilateral motor network damage (12). Conversely, increased connectivity within ipsilesional regions may reflect compensatory plasticity. In state 2, MSP patients exhibited higher connectivity in the contralesional angular gyrus, MOF, SFG, and ipsilesional SPL, suggesting better preservation of interhemispheric integration relative to SSP. State 3 revealed more complex patterns: MSP patients showed higher connectivity in the ipsilesional SMA, STP, and thalamus, but lower connectivity in the ipsilesional angular gyrus, precuneus, and contralesional gyrus rectus, MTG, and cerebellum. These results suggest greater network reorganization and motor adaptation in MSP during this state (4), demonstrating a meaningful relationship between dFC patterns and UL-FMA scores (51).

Our study introduces several innovations by integrating rs-fMRI-derived dFC with deep learning for stroke severity classification. However, it has limitations. Despite augmenting sample diversity through the sliding window approach (209 time points per subject), the overall dataset remains modest, limiting the application of end-to-end learning approaches, static functional connectivity-based 3D-CNN classification, and regression models for predicting actual UL-FMA scores. External validation is also lacking. Future studies should expand sample sizes and replicate findings in independent cohorts. Additionally, deep learning models inherently reduce interpretability. Although k-means clustering helped identify salient dFC differences between MSP and SSP, it does not fully reveal how individual features contribute to network decisions. Explainable deep learning techniques should be employed in future work to visualize influential 3D dFC features. Furthermore, our study focused exclusively on dFC; complementary approaches-such as joint time-frequency analysis and dynamic graph theory-may provide additional insights into time-varying connectivity patterns (20, 52). Future research should integrate these methods to build multi-modal representations and improve classification accuracy. Although the 3D-CNN provides an end-to-end approach without additional feature extraction, comparisons with conventional machine learning remain important once feature extraction methods are fully developed.

In conclusion, rs-fMRI-derived dFC combined with 3D-CNN offers a reliable, objective tool for assessing UL motor impairment severity in stroke. This approach may complement clinical scales such as the UL-FMA, particularly in settings with limited access to experienced rehabilitation specialists. In the future, our framework may provide a practical means to explore functional connectivity patterns associated with motor impairment severity under specific resting-state configurations.

## Data Availability

The raw data supporting the conclusions of this article will be made available by the authors, without undue reservation.

## Footnotes

^1^ www.mricro.com

^2^ http://www.fil.ion.ucl.ac.uk/spm

^3^ http://rfmri.org/dpabi
